# Enhancing the implementation of provider-to-provider telehealth in rural and remote areas: A mixed methods study protocol

**DOI:** 10.1177/20552076241242790

**Published:** 2024-04-01

**Authors:** Kaylie Toll, Joanna C Moullin, Stephen Andrew, Aled Williams, Richard Varhol, Timothy A Carey, Suzanne Robinson

**Affiliations:** 1 School of Population Health, Faculty of Health Sciences, 168274 Curtin University, Perth, Western Australia, Australia; 2 enAble Institute, 1649 Curtin University, Perth, Western Australia, Australia; 3 WA Country Health Service, Command Centre, Perth, Western Australia, Australia; 4 Deakin Health Economics, Institute for Health Transformation, School of Health and Social Development, Faculty of Health, 2104 Deakin University, Geelong, Victoria, Australia

**Keywords:** Digital health < general, telehealth < general, telemedicine < general, emergency medicine < medicine, mixed methods < studies

## Abstract

**Background:**

Virtual healthcare solutions are proposed as a way to combat the inequity of access to healthcare in rural and remote areas, and to better support the front-line providers who work in these areas. Rural provider-to-provider telehealth (RPPT) connects rural and remote clinicians to a ‘hub’ of healthcare specialists who can increase access to emergency and specialised healthcare via an integrated model. Reported benefits for the place-based provider include enhanced knowledge, expanded professional development opportunities, improved scope of practice, and increased confidence in treating more complex cases. These reported benefits could have implications for supporting and futureproofing our health workforce in terms of productivity, burnout, recruitment, and retention.

**Methods:**

The research uses an explanatory sequential mixed methods approach across multiple phases to evaluate the current implementation of Western Australia Country Health Service's (WACHS) Command Centre (CC) services and explore factors associated with their differential use. The primary population of interest and participants in this study are the place-based providers in country Western Australia (WA). Patient data constitutes the secondary population, informing the access and reach of CC services into country WA. Data collection will include service data, an online survey, and semi-structured interviews with the primary population. The data will be interpreted to inform evidence-based strategies and recommendations to improve the implementation and sustainment of RPPT.

**Discussion:**

Innovative and sustained workforce models and solutions are needed globally. Virtual healthcare, including provider-to-provider models, demonstrate potential, especially in rural and remote areas, designed to increase access to specialised expertise for patients and to support the local workforce. This research will generate new data around behaviour, perceptions, and value from the WACHS rural and remote workforce about provider-to-provider telehealth, to explore the implementation and investigate strategies for the long-term sustainment of RPPT services.

## Background

People living in rural and remote areas consistently experience poorer health outcomes compared to people living in urban or metropolitan areas.^1^ Remoteness is linked to poorer health outcomes and a higher burden of disease largely driven by reduced access to healthcare.^1,2^ Access to health services in rural and remote areas is not only a challenge of physical distance, but the demand for services regularly outstrip supply.^3^ This is partly due to a workforce shortage, a problem experienced globally.^2^ Workforce shortages and high staff turnover also impact the effectiveness of services, with research showing an association between workforce issues and a reduction in patient health outcomes, significantly higher hospitalisation rates, and decreased continuity of care.^4,5^ The workforce shortages in Australia are projected to progressively intensify over the next 40 years, partly due to an ageing population reducing the available labour force.^6^ Modelling based on Commonwealth of Australia data has estimated that the workforce needs to be four times more productive in terms of patient throughput and efficiency to meet future demand.^7^

### Virtual health solutions

Proposed as a way forward, virtual health solutions aim to build, strengthen, and futureproof our healthcare system.^8,9^ Virtual health solutions, encompassing the terms telehealth and telemedicine, have been relied on for decades in rural and remote areas as a way to increase access to healthcare due to the geographical distances and poor economies of scale.^10^ Recently, there has been a global acceleration and innovation in virtual healthcare, largely due to COVID-19 pandemic, including a rapid rollout of services that can complement face-to-face options.^11^ This has been seen as beneficial for both consumers and the health system but has emerged spontaneously and opportunistically rather than rigorously and systematically.^12,13^ For consumers, research suggests one size does not fit all. While there are many aspects of virtual healthcare that are valued, such as having choice and flexibility, consumers generally do not want virtual health to replace face-to-face services.^14,15^ In terms of the health system, there are benefits in productivity through the optimisation of staff time, including less need for travel, enabling a clinician to convert travel time to clinical time, especially in rural and remote areas.^16^ Less travel also translates to lower costs to both the provider and patient. The main benefit of virtual healthcare is providing access to services where they were previously not available. However, an increase in access has the potential for excess use and therefore not necessarily a reduction in costs.^16^

On the contrary, there is commentary about the unintended consequences of virtual health. This includes the risk of an increase in the digital divide for older and vulnerable populations, a further fragmentation of services, and concerns virtual health will replace face-to-face services, increasing local workforce shortages.^11,17,18^ Given these considerations, research on the effectiveness, value, and acceptance of virtual services is necessary to build an evidence base of virtual healthcare options so that a more systematic and strategic approach to integrated healthcare can be implemented.

### Rural provider-to-provider telehealth

Rural provider-to-provider telehealth (RPPT) services commonly involve connecting rural front-line providers (clinicians) to a ‘hub’ of urban specialists. Previous authors have defined RPPT as ‘any form of interactive support using telecommunications technology provided to health care professionals while they are caring for rural patients and populations’.^19^ The aim of RPPT is to increase access to healthcare that would otherwise be unavailable. RPPT services are comprised of virtual patient assessments, clinical consultations, mentoring, supervision, and continuing education between healthcare professionals in the inpatient, outpatient, and emergency settings.^19,20^ Totten et al.^19^ conducted a systematic review of RPPT services looking at the uptake of services, the effectiveness of the services, the barriers and facilitators to implementation and sustainability, and recommendations for strengthening future research. The review found a growing use globally for RPPT, and patient health outcomes which were similar or better when compared to no virtual health support.^19,21^ Barriers and facilitators were identified to the implementation of RPPT, including well-functioning technology, sufficient resources, and adequate payment as facilitators, and a lack of understanding of the rural context as a barrier.^19,21^ Methodological weaknesses were found in the studies examined, including less rigorous designs susceptible to bias, small sample sizes, an absence of program theory and analytical frameworks, and a lack of clearly described research questions.^19,21^

The Totten et al.^19^ systematic review reports benefits for front-line clinicians receiving virtual education and mentoring.^22^ These included improved knowledge, skills, and confidence, increased professional development opportunities, increased scope of practice, and improved safety and quality outcomes.^19^ It is not known if these benefits translate to decreased burnout, workforce turnover or improved retention. It is suggested that RPPT can decrease the total cost of care, but it is not known to what extent. A scoping review by Snoswell et al.^16^ recommends that any implementation of virtual health services should be motivated by aspects other than cost reduction. The Totten et al.^19^ review, and following workshop convened by the National Institutes of Health in the United States,^20^ identified key knowledge gaps and specific recommendations to build evidence on the emerging use and effects of RPPT. Highlighted was the inconsistent evaluation, implementation, and sustainment of these services, recommending that conceptual frameworks be used, and contextual factors be considered. Additionally, Wakefield et al.^20^ calls for new research efforts to understand the value and effects these services deliver, with an aim to improve health outcomes for rural and remote patients, by supporting the local workforce and improving access to healthcare.

This research intends to strengthen the evidence base of RPPT by providing methodologically sound research to fill knowledge gaps and recommendations identified by Totten et al.^19^ and Wakefield et al.^20^ Recommendations identified by these authors^20^ that this research intends to cover include:

- The variation of uptake across diverse settings, organisational structures, and provider types.
- The standardisation of measures across multiple sites to assess outcomes and impact.
- Evaluate whether RPPT impacts rural provider recruitment and retention.
- Engage providers to shape and evaluate RPPT to better meet community and population needs.
- Examine sociocultural factors that may influence uptake, outcomes, and sustainability.
- Use statistical approaches to minimise selection bias and address confounding effects.
- Employ mixed methodologies to triangulate qualitative and quantitative results.

Specifically, this research will utilise the Western Australia Country Health Service (WACHS) Command Centre (CC) as an example of RPPT , to assess the differential implementation of RPPT across over 100 sites in country Western Australia (WA). The CC operates 24 h a day, 7 days a week, specialising in emergency, acute, and specialty clinical services, and staffed by clinical and non-clinical staff who support over 6000 front-line providers in country WA.^23^ The purpose of the CC is to virtually support the delivery of, and equitable access to, high-quality healthcare for people in country WA, and to complement place-based service provision.

Therefore, the overall aim of this research is to increase the understanding of provider-to-provider telehealth in rural and remote settings, including its value, benefit, and sustainment, in the WACHS.

### Objectives

1. Evaluate the implementation of RPPT in country WA.
2. Explore factors associated with the differential use of RPPT in country WA.
3. Triangulate and synthesise results to develop evidence-based recommendations to improve the implementation and sustainment of RPPT.

## Methods

### Setting

WACHS delivers healthcare services to over 550,000 people across 2.5 million square kilometres.^24,25^ There are 103 WACHS-run health facilities, including six large regional hospitals, 15 medium-sized district hospitals, 48 small hospitals, and 34 health centres and nursing posts across seven geographically dispersed regions. There are additional non-WACHS health facilities and Aboriginal Medical Services that are outside the scope of this research.

**Figure 1.**
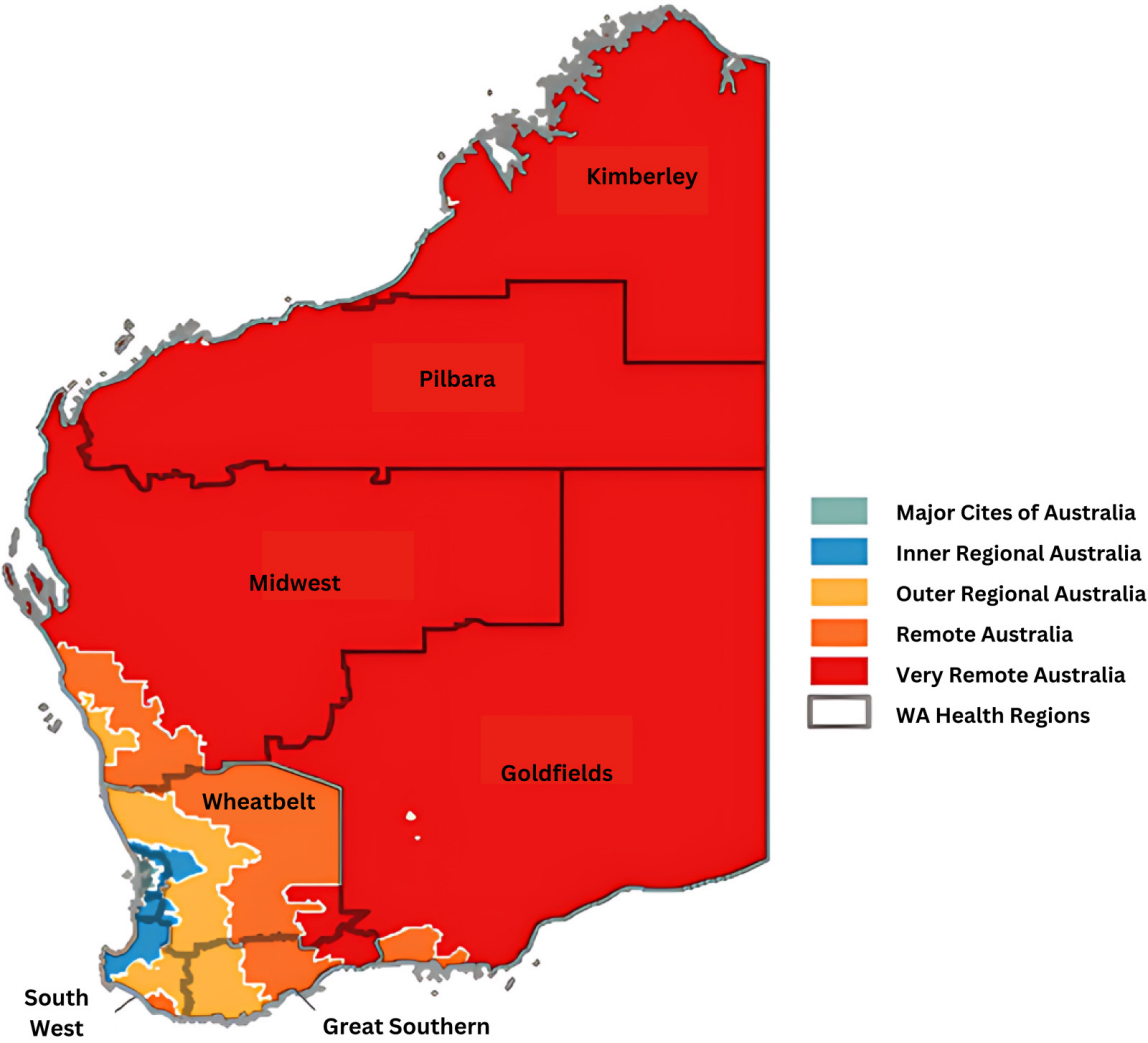
WA country health service geographical service delivery area, by region and remoteness. WA: Western Australia.

The majority of smaller health facilities are operated by generalist nurses, with medical cover provided by a local general practitioner. Each region is unique in its population, geography, health needs, and service availability, and all classified as rural and remote according to the Australian Statistical Geography Standard (ASGS) Remoteness Structure.^1^ The ASGS is based on the road distances people have to travel for services.^1^ Figure 1 shows the seven WACHS regions, overlayed with the measure of remoteness.

### Model of service

The WACHS CC began operations in 2012 as the Emergency Telehealth Service (ETS), a virtual health solution to support diagnosing, treating, and managing patients in local regions.^26^ The ‘Command Centre’ was officially launched in 2019 from recommendations of the Department of Health, Western Australia's Sustainable Health Review.^27^ A suite of services have since been implemented, beginning with the Inpatient Telehealth Service, and Mental Health Emergency Telehealth Service, and more recently, the Midwifery and Obstetrics Emergency Telehealth Service,^28^ and the Palliative Care Afterhours Telehealth Service.^29,30^ While these services differ in their functionality and purpose, they all deliver provider-to-provider specialist and clinical advice via virtual means, supporting the front-line clinicians caring for patients in rural and remote areas. The CC's Clinical services aims are as follows:

- Facilitate appropriate decision making in the treatment and care of country patients.
- Support our place-based workforce by providing guidance, advice, and access to specialist knowledge to help provide the best care possible for rural and remote patients.
- Build a collaborative, integrated workforce, blending place-, and remote-based clinicians to deliver care to the highest possible quality and safety standard and in the most culturally appropriate way.

Unlike other virtual or telehealth care, which is often between the patient and provider, these services are usually between the rural and remote front-line provider, with the patient present, and the specialist provider. The suite of CC services is enabled in 87 of the 103 health facilities.

### Population

While the objective of the CC is to improve healthcare for the rural and remote population, the primary population for this research is the direct receivers of the CC services, the approximately 6000 place-based health professionals in country WA. This includes nurses, midwives, medical officers, and medical practitioners who work across WACHS health facilities. Allied health professionals are not in scope for this research. The secondary population is the patients attending these rural and remote health facilities, often in emergency or acute situations. Patient ‘service contacts’ represent the volume and characteristics of activity at each health facility and are crucial to assessing implementation status. There has been approximately 200,000 service contacts across the suite of services since 2012.

### Design

**Figure 2.**
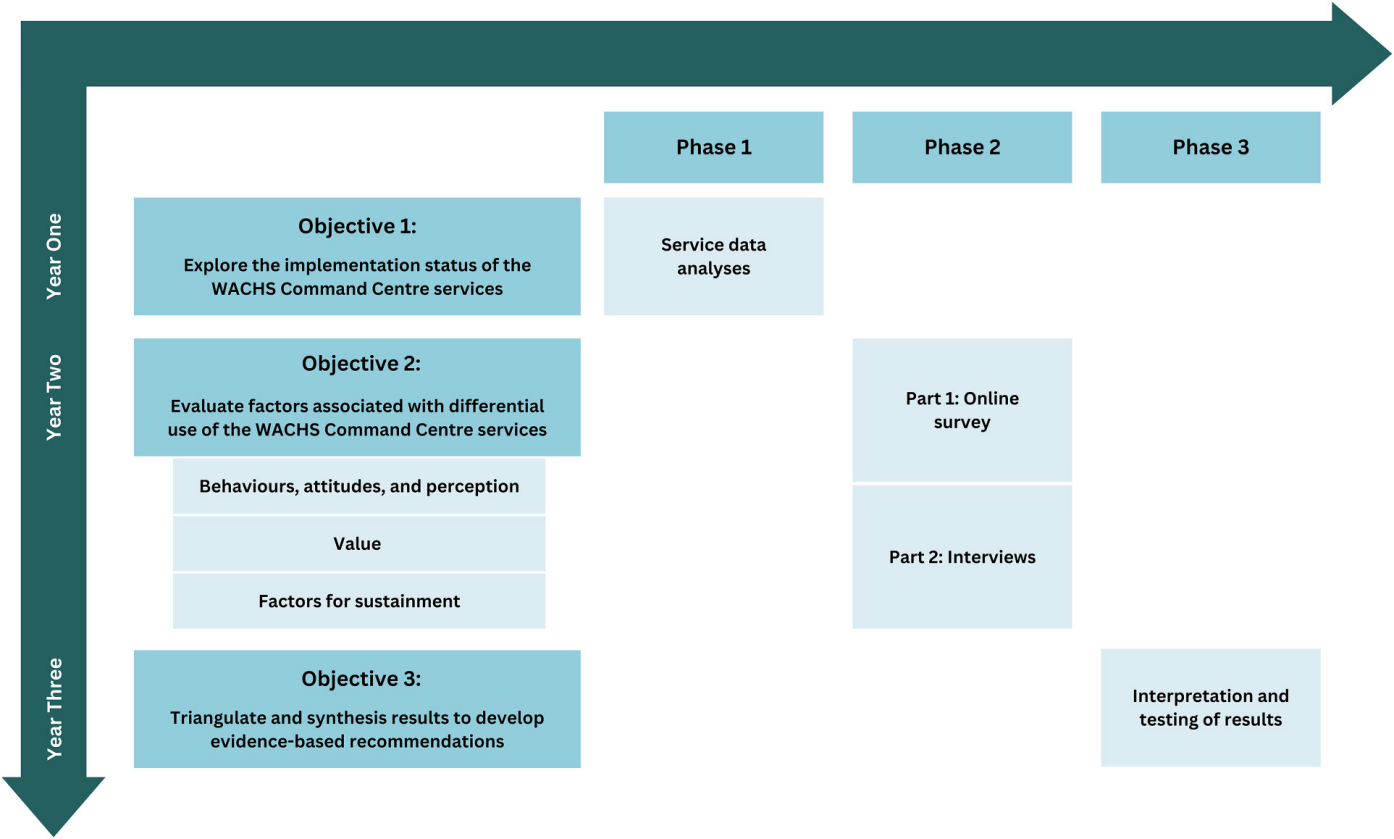
Research design.

The research will use an explanatory sequential mixed methods approach^31^ across multiple phases, displayed in Figure 2. This type of design is suitable for a real-life complex healthcare service, allowing greater understanding from multi-faceted sources.^32^ The CC is an active and dynamic set of healthcare services that have been implemented for varying lengths of time, and are operating within a larger public health system. The study will begin with quantitative data collection and analyses, followed by a qualitative phase used to explain and better understand the quantitative results.^31^ This will be followed by an interpretative phase where data are triangulated and synthesised to develop evidence-based recommendations for implementation improvements.

An Advisory Research Group will be formed at the formative stage of this research, with participants from key areas of WACHS, including representation from (a) the CC, (b) Research, Innovation and Development, (c) Strategy and Change, (d) Data Analytics, (e) provider/s from rural and remote areas, (f) health service consumers, and (g) the Research Team. This group will meet up to four times per year with a role to provide advice, feedback, and appropriateness of research components.

### Proposed phases, data collection, and analyses

There are three phases for this research: CC service data analyses; a survey and semi-structured interviews; and an interpretation and testing of results phase. These are intended to answer the research questions outlined in Table 1, mapped to research objectives. Data to be collected and analysed are described in Table 2, mapped to the expanded reach, effectiveness, adoption, implementation, maintenance (RE-AIM), and implementation outcomes framework (IOF) framework definitions defined by Reilly et al.^33^

**Table 1.** Research questions mapped to objectives.

Objective	Research questions
1. Evaluate the implementation of rural provider-to-provider telehealth services in country WA.	What is the reach and adoption of Command Centre services over time? What are the differences in reach and adoption across regions, sites, and health facility types? What are the differences in characteristics of patients involved in an episode of care from Command Centre services? What proportion of WACHS hospital presentations are referred to the Command Centre?
2. Explore factors associated with the differential use of rural provider-to-provider telehealth.	What are the differing behaviours, attitudes, and perceptions front-line providers have for Command Centre services? Is there a difference in the characteristics/demographics of providers who use Command Centre services? What do providers see as the benefit and value of the Command Centre services? What are the perceived risks to the place-based provider in using Command Centre services? What are the main reasons providers do not use Command Centre services? Did environmental or policy changes (system/outer context factors) influence service reach and adoption?
3. Triangulate and synthesise results to develop evidence-based recommendations to improve the implementation and sustainment of rural provider-to-provider telehealth.	What are the core components of Command Centre services? What adaptations have been made to Command Centre services to better suit local contexts? What factors potentially influence the sustainment of the Command Centre? How can the Command Centre services be improved to better integrate virtual healthcare and support the place-based workforce?

WACHS: Western Australia Country Health Service; WA: Western Australia.

**Table 2.** Data measures guided by the expanded RE-AIM and IOF.^33^

RE-AIM dimension	Expanded RE-AIM & IOF definition	Data measure	Data collection
Reach	Number of individuals exposed to the intervention	Number of CC patient contacts (by site, health facility type, region) for each command centre service	Service data
	Proportion of intended audience who participate in the intervention	Proportion of CC patient contacts compared to total health facility presentations	Service data
	Representativeness of individuals relative to the intended population	CC Patient contact characteristics (age group, sex, postcode, Aboriginal and/or Torres Strait Islander status, priority category, diagnosis)	Service data
Effectiveness	The degree to which the intervention is producing its intended effects while assessing potential unintended consequences and changes in quality of life	Degree to which staff agree that the intended objectives of the CC are being met	Survey Interviews
Adoption	Number of settings that participate in or are exposed to the intervention. (Organisational level)	Number of sites where CC services and infrastructure have been implemented (by health facility type and region) for each CC service	Service data
	Proportion of the intended settings who deliver or are exposed to the public health intervention. (Organisational level)	Number of sites actively using CC services compared to where services and infrastructure have been implemented by not being actively used. Determined by patient volume.	Service data
	Representativeness of settings relative to the intended population who participate in or are exposed to the public health intervention. (Provider level)	Number and characteristics of front-line providers using the CC (by site, health facility type, region) for each service	Survey
	Antecedent assessment of service recipient perception (Provider level)	Front-line provider attitudes and perceptions of CC services Normalisation MeAsure Development (NoMAD) questionnaire^39^	Interviews Survey
Implementation	Consistency of delivery as intended and in the time required across staff and organisations	Fidelity: Rollout of services according to implementation plan	Service data Interviews Project initiation documents Operational guidelines
	Adaptation Assessing indicators of adaptation prior to, during, and following implementation of the intervention	Local adaptations Cultural adaptations	Survey Interviews Project initiation documents Operational guidelines
Maintenance (individual level)	The extent to which the intervention's primary outcome is sustained ≥6 months	Provider REport of Sustainment Scale^40^	Service data Survey
Maintenance (organisational level)	The public health intervention becomes institutionalised or part of the routine organisational practices and policies	Reach and adoption over time Normalisation MeAsure Development (NoMAD) questionnaire^39^	Service data Survey

RE-AIM: reach, effectiveness, adoption, implementation, maintenance; IOF: implementation outcomes framework.

### Theoretical frameworks

An Implementation Science lens will be employed in this research. The expanded RE-AIM framework will be used, which combines the practical, robust, implementation, and sustainability model (PRISM),^34^ and the IOF.^35^ RE-AIM is a planning and evaluation framework used to assess impact of an intervention at the individual and organisational level for the indicators of reach, effectiveness, adoption, implementation, and maintenance.^36^ PRISM was developed to assess contextual factors affecting RE-AIM. This includes the intervention characteristics; perceptions and characteristics of individuals and the organisation; implementation and sustainability infrastructure; and the external environment.^37^ The IOF includes eight implementation outcomes which can influence the success of an intervention: Acceptability; Adoption; Appropriateness; Costs; Feasibility; Fidelity; Penetration; and Sustainability. Reilly, Kennedy^33^ mapped these implementation outcomes to the RE-AIM indicators, with working definitions. The enhanced mapping of implementation outcomes to RE-AIM indicators will be used to guide data collection, analysis, and the triangulation of results. This will inform how multi-level contextual factors may influence implementation outcomes, and therefore service outcomes, including efficiency, safety, effectiveness, equity, patient-centredness, and timeliness, which are all indicators of success for the intervention.^35^ Analyses of these indicators will be used to inform evidence-based recommendations to improve the implementation and sustainment of this model of care in the long term.^38^

#### Phase 1: command centre service data analyses

Data from each of the CC services will be collated from the date of implementation, or when useful data collection commenced, to the date of data extraction. These vary based on the service, with dates to be outlined on analyses.

Variables to be collected and analysed for each service are framed around the evaluation of implementation status and implementation outcomes, integral and proceeding to successful service and clinical outcomes.^35^ Service data will be collected for all ‘service contacts’ across the 103 WACHS-run health facilities, which were referred to the CC. No individual staff data are collected in the service data, therefore ‘Episode’ volume will be used to demonstrate the reach (patient level) and adoption (organisational level), as outlined in Table 2.

CC service data will be analysed via descriptive statistics including measures of frequency, percentages, central tendency, and variation. The analyses will describe each CC service, by site, by region, and by health facility type. Prior to analyses, the data will be transformed into a suitable format across the services to enable cross-tabulation across variables. The data will also be checked for missing or not applicable data or the presence of any anomalies. Inferential statistics will be used to test for analysis of variance between patient contacts (dependent variable) and the region, health establishment type, and site (Independent variables), per service.^41^

#### Phase 2: survey and interviews

Phase 2 will analyse what drives the differential usage of WACHS CC services across country WA by employing a cross-sectional survey and follow-up individual interviews, as part 1 and 2, to explore the behaviours, attitudes, and perspectives that front-line clinicians have for CC services, the reasons behind the differential use of services and contextual factors that may influence use. This also aims to elicit values and benefits front-line clinicians perceive when accessing CC services, and thoughts about improvements for future use.

#### Phase 2, part 1: survey

##### Survey instrument

Survey items, data collection, and analysis will be structured around the expanded RE-AIM and PRISM guidance,^42^ incorporating the normalisation process theory (NPT),^43^ and the Implementability of Healthcare Interventions.^44^

The survey will be divided into four parts:

1. Demographics
2. Use of CC services.
3. Perceptions (appropriateness, acceptability, and feasibility),^45^ behaviours, attitudes, and value towards using CC services.^39^
4. Staff perception of the long-term sustainability and areas for improvement of CC services.^40^

The survey will embed the Normalisation MeAsure Development (NoMAD) instrument,^46^ which covers four theoretical constructs based on NPT.^43^ These are coherence, cognitive participation, collective action, and reflexive monitoring and will be gathered in part 3 of the survey. NPT specifically provides greater understanding to the concepts of adoption, implementation, and maintenance (sustainment) from RE-AIM.^47^ Perceived risk has been shown to be a common barrier and precedent to the acceptance and adoption of technology and virtual healthcare services.^48^ Measures of risk to be included are based on Cunningham's^49^ major dimensions of perceived risk, plus additional categories appropriate to technology and service adoption.^48^ These categories are financial, performance, technological, psychological, social, physical, privacy, provider, and time. The three-item provider report of sustainment scale^40^ will be integrated into part 4 of the survey to assess sustainment.

The survey will be piloted with the research advisory team and 3 to 5 CC staff to ensure comprehension and ease of response. The survey will be anonymous and confidential to increase participation and honesty,^41^ consisting of a series of close-ended questions using a Likert scale, plus open-ended questions. The survey length, content, and mode of survey dissemination will be considered to maximise response.

##### Participants, sampling, and recruitment

The survey will target the same population as phase 1, front-line providers who work in health sites across country WA. This target population is made up of nursing services and medical officers/practitioners working directly with patients, often in acute or emergency situations, totalling approximately 6000 people. The survey will target this entire population via convenience and purposive non-probability sampling; a sample size of over 362 will ensure the results will be statistically significant, with a 95% confidence level and 5% margin for error.

##### Data collection

The survey will be conducted online using Qualtrics (Qualtrics XM, Provo, UT) and distributed via the internal WACHS Global email. The survey will be open for a 30-day period, with a follow-up email reminder sent out on days 7 and 14. Additional modes of recruitment will be guided by the advisory research group, including dissemination and promotion by regional stakeholders, posters with QR codes, social media posts, and targeted email lists. Participants will be provided with a participant information sheet and consent prior to completing the survey, with the option to withdraw at any stage.

On completion of the survey, participants can indicate if they would like to be involved in the continuing research (interviews). These details will be collected in a separate database, keeping survey responses non-identifiable, accessed by the researcher only for follow-up interviews.

##### Data analysis

Survey data will be analysed using descriptive statistics, including measures of frequency, central tendency, and range of variation.^41^

#### Phase 2, part 2: semi-structured interviews

Individual interviews will be used to provide a deeper understanding of behaviours, attitudes, perceptions, and value. The interviews will be semi-structured with an interview guide developed using the expanded RE-AIM framework and NPT as theoretical basis. This interview guide will be piloted with the research advisory team and a small number of CC staff to ensure comprehension, ease of response, and flow.

##### Participants, sampling, and recruitment

Survey respondents who indicate they would like to participate in follow-up individual interviews will be contacted by the researcher via email. Sample size will be dependent on a judgement of data adequacy and representativeness across clinician groups and location by the researcher.^50–52^ In the case of a large number of respondents, the researcher will seek advice from the supervisory and advisory teams and consider alternative options, such as focus groups. Regardless, all participants will be contacted if they have expressed interest. In the case of a low number of respondents, the advisory research group will guide additional modes of recruitment via purposive, convenience, and snowball sampling methods.

##### Data collection

Interviews will be conducted via Microsoft Teams by the researcher, scheduled for 30 min, recorded, and transcribed via Teams. Participants will be provided with a participant information sheet and consent prior to commencing the interviews, with the option to withdraw at any stage. Interview transcripts will be checked by the researcher for accuracy immediately after each interview. Transcripts may contain references to names, roles or contexts that could potentially identify participants or others in the organisation. Transcripts will be carefully reviewed to remove or generalise any potentially identifying information. All reporting will be done in an aggregate or non-identifiable manner. All data and analysis will be conducted in a secure and controlled environment.

##### Data analysis

Analysis of data collected from interviews will be analysed using the NVIVO software, guided by the Braun and Clarke^51^ reflexive thematic analysis. This type of analysis is an organic, fluid, and flexible approach, including both inductive and deductive analysis. This allows a deeper engagement and understanding with the data and any contextual information to be interpreted.^53^ Data will first be analysed inductively, with coding and theme development directed by the content of the data. This research is looking to test and validate findings from phase 1 and phase 2, part 1, so refining themes will also take a deductive approach in phase 3, where analysis is then directed by existing concepts and ideas, along with the expanded RE-AIM framework and NPT. A six-phase process will guide the reflexive thematic analysis: (a) Familiarisation with the dataset; (b) Coding; (c) Generating initial themes; (d) Developing and reviewing themes; (e) Refining, defining and naming themes; and (f) Writing up.^54^ The lead researcher will undertake the open and organic coding and theming through reflexive thematic analysis, as recommended by Braun and Clarke, which differs from codebook and coding reliability thematic analysis.^51^ Inductive reflective thematic analysis removes pre-conceived ideas and themes from the data, as much as possible. The tool developed by Braun and Clarke^53^ for evaluating thematic analysis will be used as a reflective guide throughout phase 2, part 2.

#### Phase 3: interpretation and testing of results

The purpose of phase 3 is to integrate results from phases 1 and 2 to develop evidence-based strategies and recommendations for the value and sustainment of RPPT. The RE-AIM framework^34^ will be used to frame the integration of data, with two points of data integration.^55^ The first point will be post-survey where quantitative and qualitative survey data will be integrated with service data to illustrate and explain service data. The second point of integration will be post-interviews, where the qualitative interview data are integrated to determine if there is a convergence of data, plus to illustrate and explain differential use of CC services.

Results of this interpretation will be used to identify barriers and enablers, including context-specific factors to improve the implementation and sustainment of the CC services.^56^ Stakeholder feedback and participation is essential to prioritise and make implementation and optimisation systematic.^56^ Stakeholder participation will occur via a deliberative dialogue forum with WACHS CC stakeholders to deliver results of the research, plus validate any interpretations or conclusions drawn from the research. Deliberative dialogue forums have been used widely in public health and health policy research as a formal process of interactive knowledge translation and exchange, also increasing the prospect of research translation and implementation.^57^

Participants will be invited via email through purposive and convenience sampling, and advice from the research advisory group. Participants will include front-line clinicians, CC staff, WACHS executive and managerial representatives, and WACHS staff involved in virtual health policy. The forum, informed by Boyka et al.^58^ recommendations, will be delivered face-to-face by the researcher and advisory team, with optional online attendance for rural and remote participants, for a maximum of 3 hours.

The deliberative forum will follow an implementation mapping style approach as defined by Fernandez et al.^59^ Implementation mapping is a systematic process, adapted from intervention mapping, which will be used to guide the development, selection, and tailoring of strategies to improve the adoption, implementation, and maintenance of CC services.^59^ Implementation mapping will follow a five-step process, mapped to the phases of this research:

1. Conduct an implementation needs assessment and identify program adopters and implementers.
  - Phase 1 and 2
2. State adoption and implementation outcomes and performance objectives, identify determinants, and create matrices of change objectives.
  - Phase 2 and 3
3. Choose theoretical methods (mechanisms of change) and select or design implementation strategies.
  - Phase 3
4. Produce implementation protocols and materials.
  - Phase 3
5. Evaluate implementation outcomes.
  - Phase 3.

A final technical document will be produced for internal use with a summary of research findings, insights, tailored strategies, recommendations, and setting up of a long-term monitoring framework. The report will be easily comprehensible and provided to all interested WACHS staff.

## Discussion

This paper describes the research protocol to evaluate the current implementation of the WACHS CC, a RPPT service, to explore factors for differential usage, and provide evidence-based recommendations for continual improvement. The CC's main aims are to increase access to acute, emergency, and specialist advice to those living in rural and remote areas, and to provide an additional layer of support for the place-based health workforce. As an emerging field of research, the results will contribute to a greater understanding of RPPT.

Innovative and sustained workforce models and solutions are needed globally, with virtual healthcare demonstrating potential especially in rural and remote areas to support and strengthen the local workforce,^60–63^ but are often not addressed in workforce retention strategies or reviews.^64–66^ The research outlined in this protocol will focus on the provider aspects that may influence the use of the suite of WACHS CC services. Specifically, the research will generate new knowledge to inform more strategic implementation via greater understanding of provider behaviours, attitudes, and perceptions, plus explore if the value and sustainment of these services can assist with workforce retention into the long term.

Sustainability and sustainment are often reported as the least researched implementation outcomes.^67^ This research will assist when selecting evidence-based strategies and adaptations for continual service improvement and optimisation of provider-to-provider virtual care models. The use of theories and frameworks in this research is intended to contribute to improving implementation at the local level, but to also be generalisable to the broader national and international context, to fill knowledge gaps and increase the evidence base of RPPT.

Strengths and limitations of this research are recognised. First, a non-randomised study design is chosen, which can produce risks of bias. This could include participant bias (social desirability of responses), sampling bias (volunteer bias and convenience sampling), and confounding bias. The ROBINS-I (Risk Of Bias In Non-randomised Studies – of Interventions) tool will be used throughout this research to identify and assess possible biases, plus as guidance to reduce bias in each domain included in the tool.^68^ The mixed methods design, theoretical frameworks, and a reflexive thematic analysis technique are employed throughout research design, data collection, and data analysis. These have been shown to be useful in complex healthcare settings and long-term sustainment approaches, assisting with data interpretation and reducing bias.^69,70^ Additionally, the researcher is independent of WACHS and will make this clear in the survey and interviews, while ensuring participant confidentiality.

Second, this research is focused on the perspective of the front-line providers who work with patients in rural and remote areas, and how the services can be of benefit. Although patient episode data will be captured, which outlines the extent of implementation and access status, the consumer perspective is therefore out of scope, but recommended for future research to capture a more holistic view.

Third, the suite of clinical CC services was implemented at various times and locations, with varying data collection platforms and data fields. To combat this, data will be analysed using the same data variables, allowing for comparison across services.
